# Unraveling the Enigma: Huntington’s Disease Masquerading as Treatment-Resistant Psychosis - A Case Study

**DOI:** 10.1192/j.eurpsy.2024.1003

**Published:** 2024-08-27

**Authors:** A.-G. Zanfir, D. C. Pacale, C. Moraru, I.-S. Zamfir, G. Marian

**Affiliations:** ^1^ Prof. Dr. Alexandru Obregia Clinical Psychiatry Hospital; ^2^Faculty of Psychology and Educational Sciences, University of Bucharest, Bucharest, Romania

## Abstract

**Introduction:**

This unusual case report unfolds a complex and emblematic scenario involving the diagnosis and management of a 46-year-old patient with treatment-resistant psychiatric symptoms, eventually revealing a significant association with Huntington’s Disease (HD). The initial presentation at Obregia Hospital featured early signs of psychosis, such as mood swings, social withdrawal, and mild cognitive impairment. Despite predominant treatment with atypical antipsychotics, significant improvements remained elusive.

**Objectives:**

Our primary objectives were to document the intricate diagnostic journey, the challenges faced in managing the patient’s psychiatric symptoms, and the eventual revelation of an underlying neurological disorder, Huntington’s Disease. We aimed to emphasize the importance of a multidisciplinary approach to such complex cases.

**Methods:**

The patient’s clinical course was closely monitored, and the Positive and Negative Syndrome Scale (PANSS) was used to assess the severity of symptoms upon admission. The patient’s severe psychotic state led to involuntary hospitalization. Clinical observations pointing to an underlying neurological disorder prompted a neurology consultation and further investigations, including brain CT and MRI scans, but also genetic testing.

**Results:**

The CT scan revealed potential Huntington’s Disease evolution, while genetic testing confirmed the presence of the specific HTT mutation. Brain MRI with contrast substance highlighted characteristic Huntington’s Disease changes, such as cortical atrophy, necrosis, and substantial loss of brain tissue, particularly in the basal ganglia, cortical regions, and thalamic nuclei. The patient was hospitalized for nearly seven weeks, during which various psychiatric medications were trialed with limited success. However, a gradual increase of Trihexyphenidyl dosage, as well as a wash-up with saline solution and vitamin supplements (B1, B6, and C), was initiated. Subsequently, the introduction of oral haloperidol in gradually increasing doses led to significant improvements in psychiatric symptoms, dyskinesia, and overall functionality.

**Conclusions:**

This complex case underscores the paramount importance of a multidisciplinary approach in diagnosing and managing patients with Huntington’s Disease and concurrent psychiatric symptoms. The revelation of a confirmed Huntington’s Disease diagnosis also necessitated genetic testing for the patient’s two adult children, with the son testing positive. This case illustrates the challenges of adapting treatment strategies continuously in such multifaceted scenarios and highlights the compelling need for a collaborative and integrative approach.

**Disclosure of Interest:**

None Declared

